# Women design their own vaginal microbicide trial: Suggestions on how to improve adherence from former participants of HIV prevention trials

**DOI:** 10.1371/journal.pone.0244652

**Published:** 2021-01-07

**Authors:** Lori Miller, Neetha Morar, Saidi Kapiga, Gita Ramjee, Richard Hayes

**Affiliations:** 1 London School of Hygiene and Tropical Medicine, London, United Kingdom; 2 HIV Prevention Research Unit, Medical Research Council, Durban, South Africa; 3 Mwanza Intervention Trials Unit, Mwanza, Tanzania; 4 Aurum Institute, Durban, South Africa; Washington University in Saint Louis, UNITED STATES

## Abstract

Low adherence in vaginal microbicide clinical trials for HIV prevention has impeded interpretation of trial results and hindered evaluation of potentially efficacious HIV prevention gels. Understanding the underlying reasons why women join trials and their barriers to product use can support identification of ways to improve adherence and its reporting. Eight focus group discussion workshops were conducted with 46 former microbicide trial participants in Durban, South Africa and Mwanza, Tanzania. Participants provided feedback on why women join trials, the barriers to using study gel and reporting adherence accurately, and how clinical trial design can be improved to support better adherence and its reporting. Women join microbicide trials for a number of important reasons such as healthcare and financial reimbursement. Fear of adverse effects from the investigational product was the most important reason why participants reported not using the gel. The key reason for inaccurate reporting of gel use was fear of removal from the trial. Participants made concrete suggestions for improving microbicide trial design such as applicator use testing and real time feedback, improving education to participants about how trials answer their research questions, and improving transparency and clarity about study procedures. Participants also gave feedback on an innovative trial design with a non-randomised arm. Identifying HIV prevention products for women requires better understanding of the lives of women asked to join these trials, and application of that understanding to microbicide trial design. This study has demonstrated that participants and research teams can work collaboratively to design clinical trials that meet needs of both the research and of participants.

## Introduction

Low adherence has been the Achilles heel in trials of vaginal microbicides for HIV prevention [1–7], despite more than two decades of research. It is important to understand the underlying reasons behind this phenomenon. Greater understanding of the reasons for low adherence is important to support optimal design of future bio-behavioural clinical trials in low-resource settings, effective rollout of proven HIV prevention methods in communities, and to identify ways to improve adherence and its reporting in future microbicide trials. Due to the history of low adherence in microbicide trials, the HIV prevention field has shifted focus towards long acting methods such as vaginal rings and injectables for pre-exposure prophylaxis (PrEP). However, the need for on-demand and topical HIV prevention products has been recognised by community stakeholders and researchers alike [8,9]. Continued development of a range of HIV prevention products that can meet the varying needs of users will support optimal public health benefit for HIV prevention.

As vaginal microbicides are user-controlled products, women may or may not use these topically-applied gels according to protocol. This poses difficulty for interpreting null trial results, which may be due to lack of biological efficacy, or to low adherence. This is complicated by the fact that historically, self-reported data on adherence were used during the conduct of microbicide trials.

Two key guidance documents [10,11] which provide direction for ethical conduct of microbicide trials state the importance of community stakeholder involvement in trial design to ensure scientific quality and successful implementation. As tens of thousands of women have participated in microbicide trials, they can serve as a rich source of expertise about difficulties with adherence and its reporting and provide insights on how to address these issues in microbicide trial design. At the time this study was designed, there was limited qualitative research published to understand barriers to participants’ adherence to vaginal microbicides, and this small qualitative study sought to harness the experience of former microbicide trial participants. The purpose of this qualitative study was to engage former microbicide gel trial participants in focus group discussion workshops (FGDWs) to pragmatically understand their perspectives on how to improve adherence and its reporting in future microbicide trials.

This investigation focuses on understanding the context of the lives of women recruited to join vaginal microbicide trials. This analysis examines the underlying needs of women who participate in microbicide trials, and how those needs intersect with the underlying assumptions and objectives of microbicide trials.

## Methods

Former microbicide gel trial participants from the Microbicides Development Programme 301 (MDP 301, NCT00262106) and the Vaginal and Oral Interventions to Control the Epidemic (VOICE, NCT00705679) clinical trials participated in 8 focus group discussion workshops (FGDWs). This novel method combined focus group discussions with participatory activities and was conducted in Tongaat, South Africa and Mwanza, Tanzania in 2014.

MDP 301 was a randomised, double-blind, placebo-controlled phase III clinical trial of a coitally dependent regimen of PRO 2000 gel, conducted in South Africa, Tanzania, Uganda, and Zambia, completed in 2009 [12]. Follow up was planned for 12 months and participants were instructed to vaginally insert one applicator of gel within an hour before each act of sex. Participants attended monthly visits which included product pick up, pregnancy testing, and interviews; HIV testing and pelvic exams were scheduled four times over follow up. VOICE was a randomized, double-blind, placebo-controlled phase IIB trial of a daily use regimen of 1% Tenofovir gel (and oral pre-exposure prophylaxis–PrEP), conducted in South Africa, Uganda, and Zimbabwe, and completed in 2012 [13]. Follow-up for VOICE was planned for 12–36 months, and participants were instructed to vaginally insert one applicator of gel each day. Participants attended monthly visits which included product pick up, pregnancy testing, interviews, and HIV testing; pelvic exams were performed every six months. Participants in Tongaat attended their visits at research clinics. In Mwanza, guesthouses were rented to organize temporary “clinics” where participants could be seen on designated days.

### Recruitment to present qualitative study

In Tongaat, a randomized list of former participants was used to contact them via mobile phone. In Mwanza phone numbers were not available, thus former participants were traced using locator narratives by geographical areas, alternating between urban and peri-urban areas. Participants were traced by car, and then on foot. Former participants interested in participating were booked for a FGDW session.

#### Focus Group Discussion Workshops (FGDWs)

Four FGDWs were conducted in each location, with 46 participants total. In Tongaat two FGDWs were held with MDP 301 participants, and two with VOICE gel participants. In Mwanza all FGDWs were held with MDP 301 participants. FGDWs were organised by trial, and then by general age categories. FGDWs ranged from 2–9 participants each, with an average of about 6.

While the overall FGDWs in some cases took the entire workday, with lunch provided, data collection periods lasted approximately 2–4 hours. Modifications were made to the FGDWs in real time to adjust to each group’s dynamic. FGDWs were conducted in isiZulu in Tongaat and Swahili in Mwanza. A list of sessions is provided in Table 1.

**Table 1:** Focus Group Discussion Workshop (FGDW) sessions.

	Session name	Method	Objective
	Welcome and Introduction		Welcome participants, describe logistics for the day
1	Remembering the trial	Discussion	Help participants remember the clinical trial
2	Microbicide trial presentation: how trials answer their research question	Presentation with participation	Provide education about how trials answer their research questions so participants can understand why adherence is critical in microbicide trials, and will be better able to make helpful and honest suggestions during the workshop
3	Reasons for joining the trial and gel use	Discussion	What are the variety of reasons women join trials? How do those reasons affect adherence?
4	Feelings and needs/Clinic atmosphere	Discussion + group activity	What really matters to participants? How do they feel? What are their needs, what is important to them?
5	Research staff and participants	Role-play activity	Understand participant views of research teams and how they believe research teams view participants
6	Who is the trial for?	Discussion	What is the dynamic of the relationship between research teams and participants? How might these dynamics affect adherence? How can we improve trials?
7	Telling the truth	Staff role-play, discussion, group activity	Understanding what factors affect if participants answer honestly, what aspects of the trial or relationship with staff can be changed to improve honest reporting?
8	Design your own microbicide trial	Group activity	Provide participants with opportunity to make suggestions for how future microbicide trials should be designed

**Fig 1:**
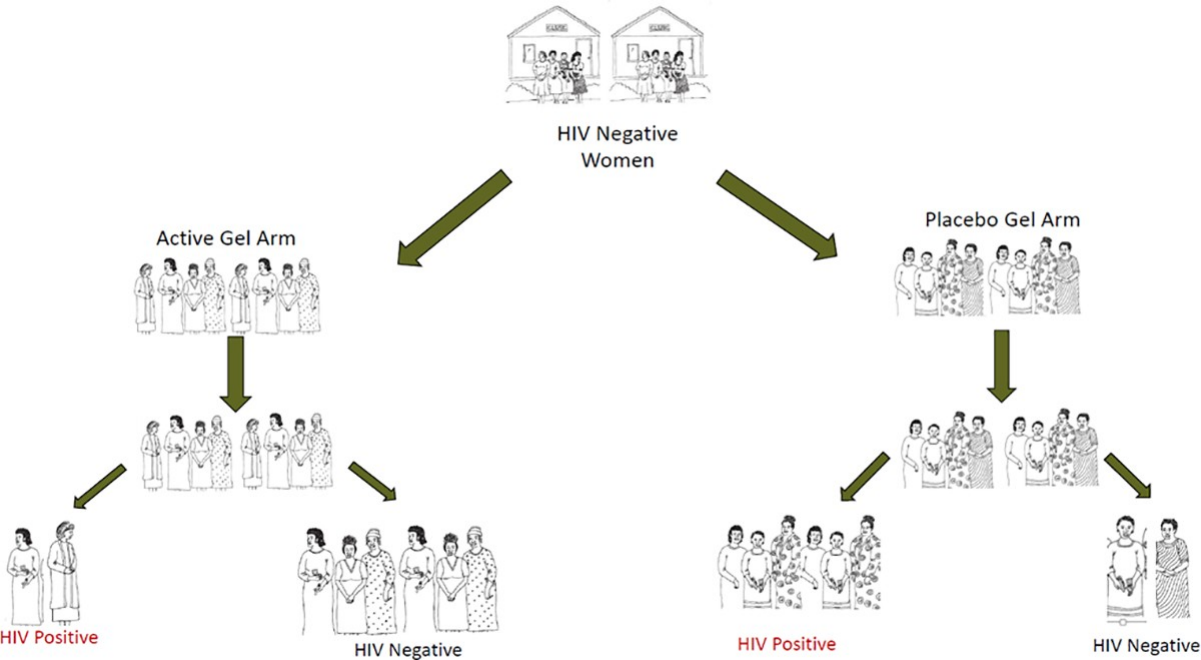
Diagram of how a microbicide trial answers its research question.

The first session began with a group discussion to remember the trial, as nearly 7 years might have passed since some had participated in the trials. So that participants could provide meaningful recommendations for future trial design, the next session included a participatory presentation, using a simple diagram, about how microbicide trials compare HIV incidence in trial arms to identify potentially efficacious products (Fig 1). The importance of adherence, and how low adherence can affect interpretation of trial results, was discussed using the Carraguard trial as an example. The Carraguard Trial was a phase III clinical trial of Carraguard Gel [14–16]. Overall, Carraguard participants self-reported using the gel at last sex act 96% of the time. However, participants were also asked to return their used gel applicators, which were tested for vaginal insertion using a special staining technique. Results of the stain assay indicated that participants used the gel about 44% of the time. The trial results were null. Ultimately, it was not clear if Carraguard was not biologically efficacious against HIV, or if participants didn’t use it enough during the trial. During this session, participants were provided with a picture of the vaginal applicator staining technique used to differentiate vaginally inserted applicators from non-inserted applicators [15].

Once it was clear that participants understood how adherence impacts the outcome of a vaginal microbicide trial such that potentially efficacious products may be discarded from the product development pipeline, the FGDWs continued with discussions and participatory activities. A discussion was facilitated to understand motivations for participation, gel use and its reporting, and how motivations for participation may have affected gel use. The next session addressed participant experience in the trials, and their feelings and needs. After a facilitated discussion, participants worked in groups to write down different feelings and needs of participants, and sort them into their own categories. Participants then engaged in a role play game to act as “research staff/researchers” and “participants” and discuss their respective attributes. The next facilitated discussion focused on views about who benefits from microbicide trials, the relationship dynamic between participants and research staff, and potential impacts on adherence and adherence reporting. The next session included a role play, conducted by FGDW facilitators, portraying different participants, their gel use, and how they reported their gel use to clinic staff. Participants were asked to reflect on the role plays and discuss what impacts a participant’s ability to be honest, and what can be changed within the trials to support participants to accurately report their adherence. The final session of the FGDWs, called “Design your own trial” involved small group work. Participants were asked to reflect on the day’s topics and then describe how they would design future microbicide trials to support participants to consistently use the gels and to feel comfortable to honestly report their gel use. At the end of the session, participants reported their suggestions back to the group.

Due to the length and intensity of the FGDWs, each workshop was facilitated by two trained individuals: one who led the discussions and one who led the participatory activities. All FGDWs were audio recorded and contemporaneous written notes were taken.

### Data processing and analysis

Each FGDW was followed by a debriefing session with staff to discuss what was said or not said, what worked, and what might need to be modified for the next day’s FGDW. Debriefing began the initial analysis of the data.

Audio recordings of the full FGDWs were transcribed verbatim and translated by trained native speakers according to standard operating procedures. 100% of transcripts and translations were reviewed for accuracy. Applied Thematic Analysis was the method of analysis for this exploratory study [17]. Transcripts were coded inductively (using NVivo10) to identify themes which emerged from the data by the principal investigator who was present at each FGDW. No coding structure was predetermined prior to analysis. The coding structure emerged and changed iteratively as meaning, in relation to the research questions, was derived from what participants said. Subthemes were added as they emerged, and structural codes were added to aid organization of the data. Negative cases, examples of views that were different than those more commonly expressed, were identified and coded when they existed. Analytic memos were used to reflect on the meaning of the quotations, differences between groups, relationships between themes, and identification of overarching themes. Codes were then adjusted and harmonised to help ensure each code described a unique concept relating to what participants said. All transcripts were coded a second time for quality assurance.

This analysis focuses on the underlying needs of participants and how those needs intersect with the assumptions and objectives of microbicide trials. A separate analysis [18], focuses on participant perspectives of how male partners influenced their adherence and suggestions from participants on how to improve adherence with regard to male partners.

### Ethics

This study was approved by the London School of Hygiene & Tropical Medicine Ethics Committee, the South African Medical Research Council Ethics Committee, the Tanzania Lake Zone Institutional Review Board and Tanzanian National Institute for Medical Research. All participants provided written informed consent.

## Results

### Participants

Forty-six women participated in this study, ranging in age from 24–73 years (Table 2). Most participants were not formally employed while trial participants. At the time of trial participation, more participants in Tongaat had attended or completed secondary school than Mwanza.

**Table 2:** Participant characteristics.

	Tongaat	Mwanza
Total Number of Participants	19	27
Number of Participants MDP 301 (*clinical trial completed in 2009)*	14	27
Number of Participants VOICE (*clinical trial completed in 2012)*	5	N/A
Age range at time of FGDW	27–51	24–73
Education level at time of trial participation		
Some/completed secondary education	15	1
Some/completed primary education	3	20
Illiterate	1	6
Employment at time of trial participation		
No work	16	4
Employed	3	0
Informal vendor	0	17
Hotel worker	0	6
Relationship status at time of trial participation		
In relationship	18	26
Single	1	1

### What underlying needs motivate women to join and stay in trials?

Participants in each FGDW brought up the health benefits of trial participation. They appreciated having their health checked on a regular basis, confidential and free testing and treatment of sexually transmitted infections, and health education. They reported feeling happy to learn about their health and how to keep themselves healthy, which motivated them to take care of themselves. Some participants noted the habit of checking their health and caring for themselves was learned during the trial and has stayed with them over the years. Participants repeatedly mentioned that money, received as reimbursement for participation, was a reason why women join and stay in trials, and how reimbursements provided a way to purchase food and necessities. Many discussed how they felt genuinely cared for by trial staff, found participation to have a positive impact on their lives, and hoped another project would start so they could participate again. Participants discussed problems with male partner infidelity and refusal of condom use as a motivating factor for joining the trial and using the gels, as they needed their own protection from HIV (Quotations provided in Table 3A and 3B, and below, with age and location of each participant).

**Table 3:** Additional quotations from participants.

**What underlying needs motivate women to join and stay in trials?**	
A: Health care & financial support	Mainly this project tempted us to check our health, it attracted us to get that little income and to make us start our businesses, therefore we profited health wise and economically. 60, Mwanza … I felt it was right in the study…knowing my status was also encouraging. . . how to behave because things are bad outside. I like it most because they were checking for diseases on us…It also encouraged us not to have unplanned babies…I liked it because I found friends. Even when you came with a problem you would be able to discuss with people. You can see that, you will leave with no problem. I also liked money, money is part and parcel [laughs]. 42, Tongaat
B: Protection from HIV	The man may have had sex outside there with three or even four women, he will just force you to have sex with him; therefore when you get the gel, maybe a little gel, you will at least be trusting yourself. 54, Mwanza
**How did participants’ needs affect their choices about gel adherence and adherence reporting?**	
C: Fear of harm from investigational drug	Yes. They are going to check it on us fools, why are they not checking it on themselves? Tongaat participant
D: Not using gel as directed due to fear of harm	The gel was not used in a big quantity because others were afraid that if she used the gel, it might bring her adverse effects. 44, Mwanza I do not think women are using the gel. When I heard about the study I heard about it from my friend … I even went to her house for her to show me that product that she takes it and put it in the toilet. She is not using it. 27, Tongaat I can say that some were not using it saying that they are scared that they are going to get sick, they are going to have diseases in the [womb], they do not know what it does when stuck in there, yes only a few using it. 38, Tongaat
E: Fear of being removed from trial and losing perceived benefits	The reason that made the participants not to say the truth, she sees that if she is chased away [removed from the trial] she will miss that allowance, because there is that allowance which is being given; if she will say the truth she will be chased away and she will not get that money, therefore she tells lies. 27, Mwanza
**Participant suggestions for improving adherence and adherence reporting**	
F: Applicator testing	In order that they may improve it I was requesting for that testing instrument, when they return those boxes… 32, Mwanza
G: Reactions to 3 arm trial design	According to my opinion maybe this will be suitable because some women don’t like the gel… if she is in this group, she can go to the group of those who don’t like the gel. 42, Mwanza …it is something that can help and make it better…these ones choose if they want the product, and you can have hope that because they chose it themselves it means…indeed it can happen that they are using it. Maybe if there can be a study like that with three groups. 27, Tongaat …If you say that they should choose, most of them will go where there is no gel. 32, Mwanza
H: Need for improved clarity	They should be given sufficient education like how we got education [today]. 43, Mwanza Yes research workers should be given more information so they can give more education to their participants. 27, Mwanza

This project has helped many people because you were being tested and if you were found with a disease they treated you…if you are at home they phone you, they come to visit you and they bring to you other necessities.

30, Mwanza

It was very nice because even if you were hungry we would see ourselves eating bread and juice even when you did not bring lunch box you were able to get something too, it was nice and get money as well when you leave. It was very nice.

42, Tongaat

### How did participants’ needs affect their choices about gel adherence and adherence reporting?

At the same time, some participants expressed fears and doubts they or others had while in the trial, including that the gel might cause them harm, due to its investigational nature. These fears indicated that they accurately understood the informed-consent process and were aware that adverse effects were possible (Table 3: C, and below).

I had fear at the beginning because they were saying they also do not know if this thing is going to work or it is not going to work… I had a fear … if it gets into my blood … in a way that is not right, and I go to them maybe having rash, … they tell me that we told you that we also do not…have a sure [certainty] about it—we are testing it on you [the product is investigational].

27, Tongaat

Participants spoke of their own worries, and rumours from community members about potential dangers of using the gels or trial participation. In particular, there were concerns about how blood from blood draws would be used.

With regard to that blood, others were claiming that why are they drawing a lot of blood from us, where is this blood being taken? Others were saying that it was being taken to [another location] to be sold, I don't know if it was true, that issue of drawing blood was bringing complexity.

30, Mwanza

In addition, participants expressed that they were not always clear on the reasons behind each trial procedure, and this raised doubts.

…they [trial staff] were taking that other blood and they were not bringing the test results, therefore they were not open, therefore it happens that you have disbelief with that place.

43, Mwanza

Trial participation met many participants’ needs for health, financial support, and feeling cared for. At the same time, some women had worries about the investigational nature of the gels, and if that might cause them harm. Lack of clarity around procedures, particularly blood draws, also raised doubts. Therefore, some participants addressed these competing needs by staying in the trial, but not necessarily using the gel as directed (Table 3: D). Participants were then confronted with a dilemma when asked to report their gel use at study visits. When asked why participants generally reported good adherence, even though they weren’t necessarily using the gel, participants stated they were afraid of being removed from the trial. This fear of losing all they valued about participation affected how some women chose to report their adherence (Table 3: E, and below).

They think they will not get the money.

44, Tongaat

Let us say they have a fear of being withdrawn and that they want to be trusted.

44, Tongaat

One participant offered another explanation for why participants might not have answered questions honestly–that some people are in the habit of not telling the truth.

… I mean it is someone's habit to speak like that, it’s like to tell lies is someone’s habit, this one has become used to telling lies and they succeed …

41, Mwanza

### Participant suggestions for improving adherence and adherence reporting

A list of suggestions which emerged from the FGDWs is provided in Table 4.

**Table 4:** Participant initiated suggestions from improving future microbicide trial design.

Participant initiated suggestions for improving future microbicide trial design	
1	Provide participants with an explanation of how a microbicide trial answers its research question, using simple terms and easy to understand diagrams
2	Provide participants with an explanation of why adherence is important in the context of a microbicide trial answering its research question
3	Provide participants with a simple and clear explanation of the investigational nature of the product, where it has been tested before, and why it is necessary to test in humans in this trial
4	Offer seminars to revisit the above topics, which can be held in waiting rooms
5	Engage experienced participants to be ‘ambassadors’ to share their experiences with other participants who may have questions about participation or experience difficulties
6	Offer ways to engage participants' male partners to learn about the purpose of the trial, the investigational gel, blood draws, and study procedures
7	Provide participants with clear information at each blood draw about the type of testing to be conducted and when the results will be provided
8	Provide participants and invited guests with opportunities to visit clinic laboratories (and other areas) to learn about the trial
9	Use a method to test applicators for vaginal insertion, and provide feedback to participants on their adherence results over follow-up

#### Applicator test and real-time feedback

While the example of Carraguard’s applicator stain test was used at the beginning of the workshops to explain why low adherence can interfere with interpretation of null trial results, this technology was not intended to be discussed further. During FGDWs, applicator testing was repeatedly mentioned spontaneously by participants as a viable strategy to monitor adherence. Participants stated that if trial participants can be shown their applicator test results in real time, it would encourage women to use the gel correctly and be honest in their reporting (Table 3: F, and below).

There should be an accurate testing instrument to show the gel which has been used and which has not been used.

39, Mwanza

#### Trial design which includes the needs of participants and the needs of research

**Fig 2:**
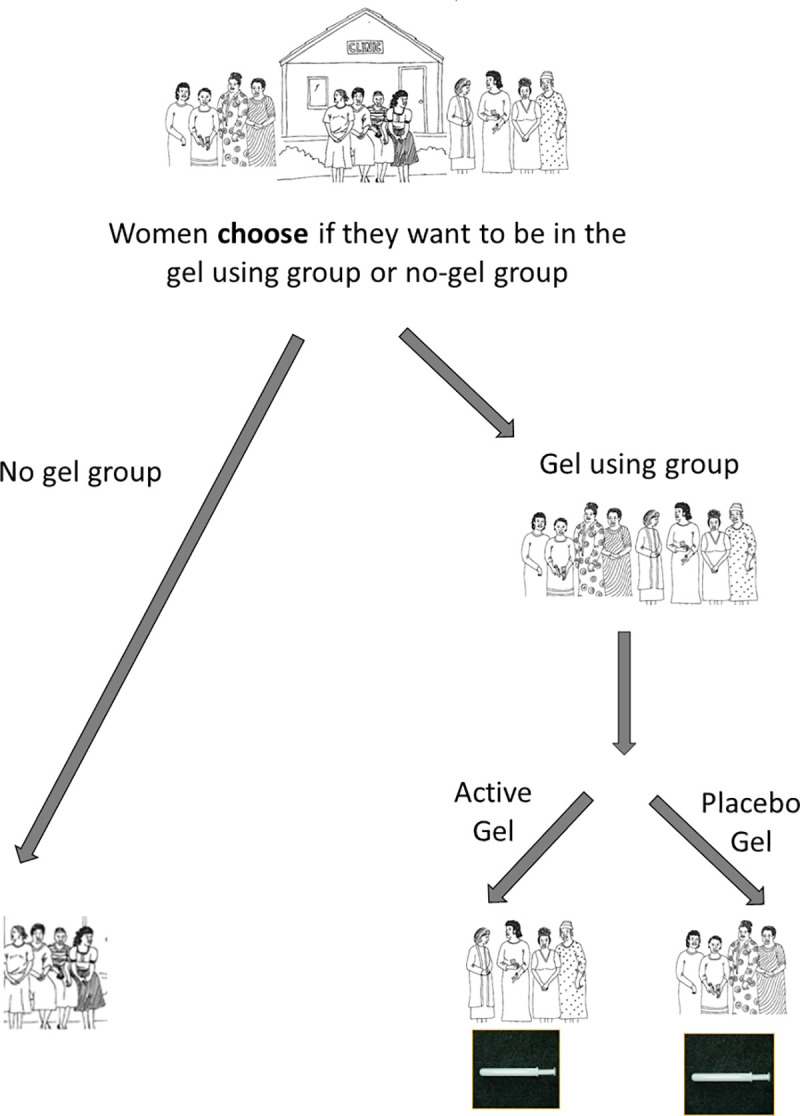
Three arm microbicide trial with non-randomised arm.

Participants were presented with an alternative trial design that included a non-randomised arm with no gel. In this hypothetical design, women who would like to join the trial choose if they would like to use the gel or not. Those wanting to use the gel would be randomised to the placebo or active gel. Those not wanting to use the gel would remain in follow-up, regularly receiving HIV prevention counselling, health testing, and treatment. This trial design was discussed via use of a simple diagram (Fig 2). Due to the initial session in the FGDWs, participant responses indicated understanding that the no-gel arm would not directly contribute data to answer the question of whether the gel works or not. Some expressed a positive response to the idea of this three-arm trial, as trial participants would be able to choose if they use the gel or not, allowing the trial better ability to ensure women randomised within the gel arms would actually be interested in using the gel (Table 3: G, and below).

It is better like this than to take it [gel] and not use it. At least it is better

because there is a group that will never use it …it is better not to take it

than to take and throw it away.

28, Tongaat

Participants who thought such a design would not be a good solution believed women joining the no-gel arm would not contribute to answering the research question of finding an HIV prevention method. They also thought that all or many women would choose the no-gel arm, demonstrating that women join the trial for reasons other than to access the candidate microbicide gel being tested for HIV prevention (Table 3: G).

#### Education about how trials answer research questions

Based on the educational session provided at the beginning of the workshop, participants noted a lack of clarity from trial staff about how participant use of microbicides would affect the outcome of the trial. They explained that as trial participants, they were not educated to understand why their adherence was critical. A desk review of participant information sheets and informed-consent materials for both trials confirmed the participants’ statements that there was no information provided which explained the relationship between adherence and study outcomes. While trial materials discussed trial procedures, randomisation, placebo, and the importance of following procedures, there was not a clear statement that HIV incidence in the two groups would be compared at the end of the trial. For individuals not trained in epidemiology, omission of this point meant participants were missing a critical piece of information to understand their use of the product and its impact on trial results (Table 3: H).

Participants discussed that if it had been explained that their lack of gel use would affect the trial’s ability to identify an efficacious product, they would have been more motivated to use the gel as directed. They suggested using the simple diagram used in the FGDWs to educate future participants to understand the process of how a clinical trial answers its research question.

#### Transparency, accuracy, clarity of information

Participants suggested that there was a need for more clarity in explaining the reasons behind procedures. When procedures were conducted in a way that was not clear, it was confusing and raised suspicions. Participants asked that procedures be explained as they were happening, so participants would know what to expect. Participants at both sites spoke about the importance of staff showing love, kindness, and care. Participants expressed the desire for them and research staff to be close. Some participants noted that when participants are treated with love and respect, fears and doubts are removed and participants feel encouraged to use the gel. More accurate and clear information was seen as a way of demonstrating transparency, respect, and love within trial conduct.

So that participants are more attracted to using the gel correctly, it is together with the researchers to be more close to the participants, they should be open to them and they should be friends…It is that they should be friends in the meaning that the researcher should not fear the participant and also me the participant should not fear the researcher so that my thoughts and hers may be close so that we may help each other.

42, Mwanza

For example, participants at both sites stated that they did not always have clarity about the use of the blood being drawn and this raised suspicions amongst participants and community members. Participants expressed that better explanations about the blood draws would have allayed their fears and addressed rumours in the communities.

… these researchers, maybe they should be going to people and give them just little seminars that when you give your blood, not that your blood is going to be sold, that blood is going to be stored in a special place and if you want to prove that it is your blood, we have the ability to take you… we go and show you that here is your blood.

39, Mwanza

## Discussion

This qualitative study used a novel method combining participatory activities with focus group discussions and endeavoured to gain insights on how to improve adherence and its reporting in microbicide trials from the perspective of former trial participants. Efforts that had been implemented to improve adherence within microbicide trials such as CAPRISA 004, VOICE, and FACTS 001 [19,20] included adopting more participant-centred approaches to adherence counselling which address barriers to adherence and tailor counselling to help participants increase gel use. While these adherence-support strategies may have shown improvements in adherence, these methods were not necessarily effective to the extent needed. The underlying assumption of these approaches is that participants enrol in microbicide trials with the intention of using the microbicides.

This analysis examines the participation of women from the perspective of the underlying needs which motivate women to join and stay in trials (male partner influence on adherence is reported separately) [18]. This study uniquely contextualised that low adherence and inaccurate adherence reporting are driven by the reality that many women in communities where microbicide trials are conducted participate in trials for a number of important reasons which are not necessarily related to interest in the study gel. Thus, there is a fundamental difference in the objectives of many women agreeing to participate in a microbicide trial and the research objectives of trial implementers. At the same time, participants want access to a female-controlled HIV prevention product, and want microbicide trials to answer their research questions, yet may not understand how their personal adherence affects trial results.

This study, as well as other vaginal microbicide and oral PrEP studies, identified a number of reasons why participants were attracted to join and stay in trials, but had concerns about the potential impacts of using study product and always reporting their adherence accurately. For many participants, the trials provided free, high-quality, confidential health services [21–25]. Participants spoke positively about being tested for health issues, receiving treatment, knowing about their health, gaining valuable education, and being cared for by trial staff. The trials, through reimbursements, provided a type of income [19,20,22,24,25]. When asked why they thought it was difficult for some participants to report gel use accurately, the most common response was participants feared they would be asked to leave the trial, thus losing access to healthcare and reimbursements [21,23]. An important reason for suboptimal gel use was fear of harm to health due to their investigational nature [22,24,26].

### Recommendations for improving adherence and adherence reporting

Participants in this study gave concrete and viable suggestions on how to improve adherence and its reporting in the context of trial design. Participants suggested two ways that staff could improve clarity of information to help improve adherence. First, they recommended use of a simple diagram and explanation to show the link between adherence and how a trial answers its research question, empowering each participant to understand how their role in using the study gel can affect the overall outcome of the trial. This explanation needs to state explicitly that the number of women who get HIV in the placebo arm and the active arm will be compared to see if the active drug reduces HIV infection. Second, participants recommended that staff provide clear explanations about each aspect of the trial as it was happening to reduce concerns that could negatively affect product adherence.

Acknowledging that participants do want an effective female-controlled method, participants suggested applicator testing and real-time feedback to participants as a way to improve adherence and its reporting. An advantage of applicator testing is that it can preserve blinding and be reported to each participant over her follow-up in real-time. Drug blood levels or other biomarker testing, in contrast, cannot be reported at an individual level due to blinding. Since the Carraguard trial, more robust technologies using biomarkers are being developed that could eliminate some of the limitations of the original applicator dye-test [27,28].

Participants gave thoughtful reactions to an innovative trial design with a non-randomised no-gel arm (Fig 2), intended to address the fundamental issue that some women join and remain in microbicide trials without using or intending to use the investigational gels. This trial design gives women an acceptable way to participate without having to pretend they are using gel. Women interested in using the gel would elect to do so and would be randomized to placebo or active gel. This design seeks to improve adherence in the gel arms by eliminating the dilution effect [4] caused by women who do not use the investigational gel. Participants in favour of this trial design thought it would be appropriate because women would have freedom to choose if they use the gel or not, would still benefit from the trial, would not waste gel unnecessarily, and would not prevent the research from discovering a potentially efficacious microbicide. While this design may improve adherence, it will certainly be more costly than a conventional trial, as more women will be enrolled. Microbicide effectiveness trials are expensive, with estimates of phase III trials costing up to $70 million USD [29,30]. If multiple trials have null results due to low adherence, considerable funds are wasted and a potentially efficacious product may be eliminated from the product development pipeline. While initially expensive, investing in a trial design that could potentially meet the needs of women in high HIV incidence communities and meet the needs of the research might be a wise investment for the long-term objectives of HIV prevention research. This design may also give some indication of product acceptability in the particular population.

### Limitations and strengths

This was a small qualitative study which asked former participants to recall their participation in past microbicide trials. For most, considerable time had passed, which could affect their ability to remember. The majority of participants had participated in the MDP 301 trial. Two contrasting locations and cultures were selected, and the unique format of FGDWs included discussion and participatory activities, addressing research questions from several angles. This study was independent of the initial trials–this, together with the time elapsed since trial participation, may have supported participants to speak more candidly. The orientation provided to participants at the outset of the FGDWs, which included information about low adherence in microbicide trials, may have influenced how participants shared their perspectives. The focus of this study was pragmatic and oriented towards problem-solving. While this study is not representative of all microbicide trial participants, views reported here add to the body of understanding of trial experiences. This was the first participatory qualitative study conducted to engage microbicide trial participants in explicitly thinking about the design of future microbicide trials to address the challenge of suboptimal adherence and inaccurate reporting.

## Conclusion

The field of microbicide research has endeavoured to discover a product women can control to protect themselves from HIV [31]. In addition to seeking new modes of microbicide delivery, such as rings [32,33], and improving adherence counselling and biomarker testing, it is important for research teams to examine and effectively address the underlying dynamics within microbicide trials. For optimal results, these underlying dynamics can be addressed in the actual design of microbicide trials. This may require thinking creatively to develop different ways of designing clinical trials. As this study has demonstrated, participants and research teams can work collaboratively to consider such designs. When participants and trial teams work together with transparency, respect, and mutual understanding [11], difficult challenges such as low adherence and inaccurate reporting can better be addressed in future trials of any intervention.

The methods used in this research, which employ participatory activities to gain a deep understanding of the context of the lives of stakeholders, and ask stakeholders to contribute to the design of projects, can be used as a model for how future research or programmatic rollout of proven HIV prevention methods can be planned.

### Dedication

We dedicate this manuscript to our dear colleague Prof. Gita Ramjee, co-author, mentor, and friend. Gita passed away unexpectedly of COVID-19 complications in March, 2020, while this paper was being finalised. We are deeply saddened by Prof. Ramjee's untimely death. She was an inspirational role model and an international renowned scientist. Gita dedicated herself tirelessly to find methods that empower women to take control of their HIV prevention and reproductive health rights through informed choices. Her death is a loss to women in Africa, to the scientific community, and to the world. Dear Gita, your work lives on through the many researchers you have touched. We miss you.

## Supporting information

S1 Appendix Focus Group Discussion Workshop (FGDW) guide. (PDF)
