# Supplementary material for: Women design their own vaginal microbicide trial: Suggestions on how to improve adherence from former participants of HIV prevention trials
Source: PLoS One. 2021 Jan 7;16(1):e0244652. doi: 10.1371/journal.pone.0244652 (PMC7790400; doi:10.1371/journal.pone.0244652)
Supplement: S1 Appendix — (PDF) [file pone.0244652.s001.pdf]

INTRODUCTON AND WELCOME

Thank you all for coming today. My name is ______.We are very happy that you came and are looking forward to hearing your opinions. As you know, your participation is confidential and we will not identify you by name. We also ask that you please not share information you have heard from other participants outside of this group to respect each other's confidentiality. Your participation is voluntary and you are free to leave any time if you wish.

The activities in the informed consent form refer to the activities going forward – which should not take more than 4 hours. The part before the research activities such as registration and informed consent also take time and that is difficult for us to predict as it depends on how many participants arrive and at what time. Also, please know that if we called you to invite you to participate today, you are eligible to take part.

The purpose of this study is to learn about your experiences as past microbicide gel trial participants and to get your ideas and suggestions about how to improve adherence to microbicide gels for future trials. As you have experience in participating in the trials, we see you as the best experts to give advice. We truly hope you will feel comfortable to give us your honest opinions. There are no right or wrong answers. Honest opinions can help improve the research and make it better for everyone.

We also want to note that you can feel free to share your own experiences or ideas, but should also feel free to tell us about what you have heard in the community, from other participants, or what other people might have said or think. Also, you do not have to speak about yourself personally.

During this discussion we want everyone to feel comfortable to speak freely and want to respect everyone's opinions and chance to speak. As you know we are recording this workshop, so it will be important that only one person speaks at once so we can hear the recording clearly. Also, for the recording, we would like to ask you to please identify yourself by your number each time you speak. If you forget, we might say it out loud for the recording.

So today we will start off with some discussions and activities, we will have a break, and then some more activities. We will have lunch together, and then more activities after lunch.

Again, we welcome you today and looking forward to hearing your opinions. We also hope you enjoy the day with us.

How is everyone feeling?

To get started, let's go around the circle and if everyone can please say which trial you participated in and for how long were you a participant. Also, please say your number when you start speaking.

DISCUSSION **REMEMBERING TRIAL**

1. What do you remember about the trial?

-What was the purpose?
-What were you asked to do?

To understand why adherence is critical in trials, so participants can make helpful and honest suggestions in the workshop

PRESENTATION WITH PARTICIPATION
MICROBICIDE TRIALS

[Hand out slides or turn on projector and display slides]

To help us remember the trials, we have a couple of pictures to share with you

- In microbicide trials, we are testing a new gel to see if it can prevent HIV
- What do you remember about the trials? Does every participant receive the same gel?
    - That's right, some women received the real drug, and some women received a drug that looked exactly the same, but didn't have the drug in it
    - Does anyone know why that is?
    - That's because it is a new drug and we don't know if it works yet. So the only way to test it is to see how it works in humans – and you must compare the new drug against no drug – or else you won't know if it the drug is able to block HIV
- The only way to test new drugs to see if they work is to do it in humans – this has to be done before it can be approved by the government to be used on humans. So all of the drugs you buy at the pharmacy or that doctors give you have been tested just like in the trial you participated in
- So in this picture we see a group of HIV negative women in a community where there is HIV
    - Then what happens?

- Yes, the women are asked to use the gel regularly

- And here, at the bottom of the picture, what can you see?
  - Yes, some women who used the placebo get HIV and some women who used the drug get HIV
  - We are looking to see if less women in the active gel group get HIV. If they did, that means the drug blocked HIV

- Now what happens if the women don't use the drug? Will we have fewer women with HIV in the active group?
  - That's right, if they don't use it, the drug can't have a chance to protect the women from HIV if it is effective, and there will be the same number of women who get HIV in both groups
  - In nearly 20 years of microbicide trials, woman reported that they used the gel most of the time
  - However, we still don't have a drug that has been approved by the government

[Move to next slide]

- This picture shows the applicator in one trial called Carraguard that was conducted here in South Africa.
- In this trial the women reported that they use the gel most of the time.
- The results of the study found that the gel did not prevent HIV
- Let's talk about these applicators used in the Carraguard trial
  - What is the difference between the two applicators? (showing picture)
  - That's right, one shows blue and one doesn't show any color
  - In this study women were asked to bring back all of their used applicators
  - In this study, they developed a special test with a dye that turns blue if the applicator has vaginal fluid on it.
  - During the study, women said they used the drug most of the time.
  - But when they tested the applicators at the end of the study, the ones that showed blue were less than half
  - In this study the women didn't use the gel very much
  - And the trial also did not show that the drug prevented HIV
  - Thus this drug was discarded as a possibility for women to protect themselves against HIV

- But in reality, it might be that the drugs works, but we just don't know
- Low use of the gel happens in many of the trials, and that is why we are here today.
- We are trying to find a way to help women protect themselves from HIV
- But we need to know more about why they don't use the gel in these studies so that can better design our trials so we can test the drugs and find one that will work and be approved by the government

What is the real motivation behind women joining the trial? How does that affect Adherence?

DISCUSSION **REASONS FOR JOINING TRIAL + GEL USE**

2. Why do women in this community join microbicide gel trials?

- Are there other reasons for joining the trial?
- Some people have told us because of better health care. What do you think about that?
- Some people have told us to know their HIV status. What do you think about that?
- Some people have told us because they want a little extra financial support. What do you think about that?
- Anything else?
- What are the most important reasons? And why?

3. How much do women use the gel?

- Tell me about the different types of gel users
- Which group do you think is most common?
- Are there women who don't want to use the gel at all?
  - How common are they?

4. How do their reasons for joining the trial affect their gel use?

- What do others think about that?
- Can you give any examples?

5. What suggestions do you have to improve adherence for these women?

- Can you explain more about that?
- For women who don't like the gel,
  - What would women think of a pill?
  - What would women think of a ring inserted in the vagina for a month?

6. ***For women who are interested in health care, but not using a product, what would they think of a study like this: [show diagram]

- At enrollment, women can choose on their own to be in a gel-using group or the no-gel using group.
  - If they choose the group not using gel, they still come to the clinic and receive the same procedures like pap smear, HIV tests, counselling, etc.
  - If they choose to be in the group using the gel, they get assigned to either the placebo gel or the active gel
    - What would women think of a study like this?
    - How might this affect their use of the gel?

What really matters to participants?

How do they actually feel?
What do they care about?

DISCUSSION **NEEDS/FEELINGS/CLINIC ATMOSPHERE**

7. Tell me about your experience being in the trial? What was it like?
- How did that make you feel?

Try and remember entering the clinic on your first day

8. What needs did you have as a new participant? (either for being a participant or in life – things that matter to you, concerns)
- Can you give an example?

9. How did you feel when you were a new participant?
- Can you give an example?

Now think about what it was like to go the clinic after you had been participating for a while

10. What were your needs once you had already been participating for a while?
- Can you give an example?

11. How did you feel coming into the clinic later in your participation?
- Can you give an example?

12. How could the microbicide trials address participants' feelings better?
- Do others have suggestions for improvements?

Thank you. Now we are going to play a short game.

ACTIVITY **NEEDS AND FEELINGS**

Please divide into two groups
[Provide participants with directions such as half on one side and half on the other]

Needs
Here are X color cards
Please think about the discussion we just had
The purpose of the game is to write down as many NEEDS (for example things you need met, concerns, things you care about) that you think participants had at the time they were participants –either for the trial or in life- in exactly 2 minutes. You will write as many participant NEEDS as you can think of.
Please write as quickly as you can.
Go!
1 minute remaining!
Stop!

Feelings
Now we will do the same thing, but now we will write down as many FEELINGS (emotions or sensations) that you can think of that participants might have had during the time they were in the trial.
Please, think about the conversation we just had around your experiences and feelings while in the trial.
You have 2 minutes to write down as many participant feelings you can think of
Please write as quickly as you can.
Go!
1 minute remaining!
Stop!

Together and Sort

Now, everybody please come together.
Let's put all of the cards here on the floor.
Now for the next few minutes, we would like you, as a group to sort the cards into any categories you see. You can do this as one group. You can use these larger blank cards to write the names of the groups or categories
Go!

2 more minutes.

Time is up!

Please describe how you sorted the needs and feelings?
[allow one person to read the categories and cards placed below]

- Why did you decide that?/What was important about that?

ACTIVITY RESEARCH STAFF/RESEARCHERS AND PARTICIPANTS

Now we are going to play a role play game
[Small pieces of paper are cut up and "participant" or "research staff/researcher" is written on each.]
[All pieces of paper are put in a bowl]

We will pass the bowl around, everyone please take one piece of paper

What did you get?
[Allow each participant to say what they got]
Good. Now, please, let's have the research staff and researchers sit on this side, and the participants sit on this side

[Give white coats/props to the researchers]

Research staff and researchers describe themselves +
Participants describe themselves

So over here, we have research staff and researchers.
And on this side we have participants.

In this first activity, we would like each group to use these cards to write down words to describe themselves. We would like you to feel free to be completely honest. There are not right or wrong answers.

So research staff and researchers, please write down on cards, as researchers, how you would describe yourselves as people? What words would you use to describe yourselves?

Participants, please write down on cards, as participants, how you would describe yourselves as people? What words would you use to describe yourselves?

You have 2 minutes! Begin!

Research staff and researchers, please read out your cards
Participants, please read out your cards

Research staff and researchers describe participants + Participants describe research staff and researchers

Now, we will again use the cards to write down words – but this time:

Research staff and researchers, we would like you to tell us what words you would use to describe participants – you can say anything at all

Participants, we would like you to tell us what words you would use to describe research staff and researchers, again please be honest, you can say anything at all

You have 2 minutes! Begin!

Research staff and researchers, please read out your cards
Participants, please read out your cards

Participants describe how they think research staff and researchers see participants

Now, for the final part, we have one more question for the participants.
Participants, do you see the research staff and researchers over there?
Can you please tell us how YOU think those research staff and researchers see you, the participants? What kind of people do you think they think you are?
Please be completely honest and include any thoughts!

You have 2 minutes! Begin!

Participants, please read use your cards.

TEA

Is the dynamic of the relationship between researchers and participants affecting adherence? If so,
How can we improve trials so women are likely to use the gel?

DISCUSSION **WHO IS THE TRIAL FOR**

[participants sit in a circle facing each other, facilitator is outside of the circle moving around]

13. Who does the trial benefit?

- Is it to help the women? Are there other ideas?

14. Is there anyone else who benefits from the trial?

- Some people have told us that research staff and researchers benefit. What do you think about that?

15. Do participants feel like they are the most important part of the trial?

- Tell me more about that

16. How do these things affect if participants use the gel?

- Does that affect participants' motivation to use the gel?
- You mentioned X…how do you think that might affect participants motivation to use the gel?
  - In what way?
- What do others think? (re-iterate some comments they made earlier)
  - Does that affect participants' motivation to use the gel? How?

You mentioned XXX affects participants' motivation to use the gel.

17. What suggestions do you have to improve the trial so that women don't experience X and could feel more motivated to use the gel?

- What are other suggestions do you have to improve motivation to use the gel?
- Are there other ideas for things to change about the trial to address this?
- What do others think?

Is there something about the dynamic between "research staff/ researchers" and participants that affects if women can speak honestly?

How can we change the trial design in a way that would improve honest reporting?

DISCUSSION
TELLING THE TRUTH

> **Staff Role Play**: Facilitators perform two vignettes demonstrating two examples of using the gel at last sex act. When asked about gel use at last sex act at the clinic, one participant answers honestly and the other does not

18. What do you think of the two role plays?

19. How difficult do you think it was for women in the trial to say they hadn't used the gel if they hadn't?

ACTIVITY **TELLING THE TRUTH**

Now we will do another activity.

In this activity you will work as one group.

Please look at this question and write as many answers as you can think of on these long cards:

What makes it difficult for participants to say they hadn't used the gel when they hadn't?

- [Was it uncomfortable to answer honestly?
- Was it to be polite?
- To please staff members?
- To please/fit in with peers?
- Because they were embarrassed?
- Because they were worried they'd done something wrong?
- Fear of criticism
- Fear they would be removed from the trial?]

Please read us your cards
[probe to have further explanation on the points]

Now we have one more question which we would you to write answers on these cards:

How can we change the trials to make women feel more comfortable to say whether they used the gel or not?

LUNCH

ACTIVITY **DESIGN YOUR OWN TRIAL TO IMPROVE ADHERENCE AND ITS REPORTING**

Please think about everything we have talked about today.

We have discussed:

- How we test microbicide gels to see if they prevent HIV (and why using the gel is important)
- Why do women join these trials?
- What were your experiences in the trial?
- What needs and feelings do trial participants have?
- How do researchers see participants?
- How do participants see research staff and researchers?
- Who do microbicide trials benefit?
- What makes it difficult for participants to honestly report their gel use to trial staff?

Remember, the purpose today is to give ideas about how to improve microbicide trials so that

- Participants are more motivated to use the gel
- Participants feel more comfortable honestly reporting their gel use

So we would like you to think about everything we have discussed and also about these trials

As participants, you are experts – you know what women liked or didn't like about the trials. You know why women choose to use the gel or not.

If you were designing a microbicide trial, and you wanted to improve adherence and honest reporting, how would you make the trials different?

What would they look like? How could we change the trials so that woman would use the gel and report honestly?

Of course, this should be a trial you would be willing to participant in.

- Please divide into two groups
- You each have flip chart paper and markers
- Here are a set of questions to guide you
- Thinking about what I have just said, and this set of questions:
- Please think about how you would design a trial better so that we can improve adherence.

- You can write down as many ideas as you like.
- You have 20 minutes to do this, and then you will present your ideas back to the other group

- 10 minutes left!

- Ok, time to finish please, let's all come together now

- Group 1 please tell us your ideas
- What do others think about those ideas?
- Group 2, please tell us your idea
- What do others think about those ideas?

- Group 1, can you please explain how X would improve adherence?
- Group 2, can you please tell us more about Y – how would that improve honest reporting or motivation to use the gel?

CLOSING

Thank you everybody. We have come to the end of our day. Thank you all very much for your ideas and your participation today. We appreciate your taking time today out of your day.

Now, does anyone have any questions they might like to ask?
Any others?

And once the study is done, how would you like to learn the results?

Okay, thank you again.
